# Single-cell sequencing and multiple machine learning algorithms to identify key T-cell differentiation gene for progression of NAFLD cirrhosis to hepatocellular carcinoma

**DOI:** 10.3389/fmolb.2024.1301099

**Published:** 2024-06-27

**Authors:** De-hua Wang, Li-hong Ye, Jing-yuan Ning, Xiao-kuan Zhang, Ting-ting Lv, Zi-jie Li, Zhi-yu Wang

**Affiliations:** ^1^ Department of Immuno-Oncology, The Fourth Hospital of Hebei Medical University, Shijiazhuang, Hebei, China; ^2^ Division of Liver Disease, The Fifth Hospital of Shijiazhuang, Hebei Medical University, Shijiazhuang, Hebei, China; ^3^ Department of Pathology, The Fifth Hospital of Shijiazhuang, Hebei Medical University, Shijiazhuang, Hebei, China; ^4^ Department of Immunology, Immunology Department of Hebei Medical University, Shijiazhuang, Hebei, China

**Keywords:** NAFLD cirrhosis, hepatocellular carcinoma, single cell, machine learning, LDHA

## Abstract

**Introduction:** Hepatocellular carcinoma (HCC), which is closely associated with chronicinflammation, is the most common liver cancer and primarily involves dysregulated immune responses in the precancerous microenvironment. Currently, most studies have been limited to HCC incidence. However, the immunopathogenic mechanisms underlying precancerous lesions remain unknown.

**Methods:** We obtained single-cell sequencing data (GSE136103) from two nonalcoholic fatty liver disease (NAFLD) cirrhosis samples and five healthy samples. Using pseudo-time analysis, we systematically identified five different T-cell differentiation states. Ten machine-learning algorithms were used in 81 combinations to integrate the frameworks and establish the best T-cell differentiation-related prognostic signature in a multi-cohort bulk transcriptome analysis.

**Results:** LDHA was considered a core gene, and the results were validated using multiple external datasets. In addition, we validated LDHA expression using immunohistochemistry and flow cytometry.

**Conclusion:** LDHA is a crucial marker gene in T cells for the progression of NAFLD cirrhosis to HCC.

## 1 Introduction

Hepatocellular carcinoma (HCC) is the sixth most prevalent form of cancer and the third most common cause of cancer-related mortality (Sung et al., 2021). In more than 80% of cases, HCC occurs on a background of liver cirrhosis (LC) (El-Serag, 2011), indicating that the precancerous environment of liver cirrhosis plays a crucial role in HCC (Affo et al., 2017).

The immune-inflammatory process is crucial for the advancement of LC and the creation of a precancerous milieu. During LC, the immune function is markedly compromised, resulting in cirrhosis-associated immune dysfunction (CAID), which is characterized by systemic inflammation and immune deficiency. CAID plays a pivotal pathophysiological role in chronic liver disease (Albillos et al., 2022). Patients with CAID are more likely to develop hepatic failure or HCC. Systemic inflammation and immunodeficiency show dynamic changes in CAID, gradually aggravating in conjunction with the progress of compensated cirrhosis without a clear critical point. In CAID, excessive production of reactive oxygen species by neutrophils induces tissue damage and fibrosis, as well as depletion of phagocytic cells (Tranah et al., 2017). Circulating CD4^+^ T cells decrease because of excessive splenic size and activation of cell death mechanisms by bacterial translocation (Lario et al., 2013). Continuous inflammation causes a gradual decline in the effector function of CD8^+^ T cells, leading to their transition into a state of T-cell exhaustion (Franco et al., 2020). The anti-inflammatory cytokine IL-10 is significantly increased (Clària et al., 2016). These represent a significant barrier to antiviral and antitumor immune responses and create a marked state of immunosuppression. The immune microenvironment in CAID plays a crucial role in suppressing immune function, promoting immune evasion, and facilitating tumorigenesis (Tsochatzis et al., 2014). However, the mechanism of how T cells promote cirrhosis or tumors in CAID remains complex and unclear.

Viral hepatitis, NAFLD, and alcoholic hepatitis are common pathogenic factors associated with LC and HCC. Among these, viral hepatitis is the main cause. However, the prevalence of viral hepatitis infections has decreased owing to the widespread availability of hepatitis B vaccines and the development of direct-acting antivirals targeting the hepatitis C virus. With the increasing incidence of obesity and diabetes attributed to pervasive lifestyle modifications, nonalcoholic fatty liver disease (NAFLD) and related HCC have emerged as prevalent chronic liver diseases globally, imposing a substantial burden on global health (Golabi et al., 2021). Therefore, it is imperative to address the urgent clinical issue of effective prevention of tumorigenesis by identifying potential immune biomarkers and therapeutic targets.

The emergence of single-cell sequencing methodologies coupled with next-generation sequencing technologies has facilitated more in-depth exploration of cellular attributes and subtypes at the individual cell level. Single-cell RNA sequencing (scRNA-seq) and transcriptome data were collected from publicly available databases for subsequent analyses. By analyzing scRNA-seq data, we identified distinct clusters of immune cells and differentiation-related genes associated with T-cell differentiation trajectories in NAFLD-related liver cirrhosis. To enhance prognostic prediction, 81 machine-learning algorithms were employed to construct prognostic signatures based on T-cell differentiation-related genes. Finally, we selected the best algorithm for calculating the risk scores. Among these signatures, lactate dehydrogenase A (LDHA) stood out as particularly promising and was systematically analyzed.

LDHA functions as a pivotal enzyme in the terminal phase of glycolysis. It is actively involved in both anerobic and aerobic glycolysis, a process known as the Warburg effect. During aerobic glycolysis, LDHA acts as an enzyme that aids in the transformation of pyruvate into lactate by oxidizing nicotinamide adenine dinucleotide dehydrogenase (NADH) to NAD+ (Ding et al., 2017). LDHA plays an essential role in tumorigenesis, metastasis, angiogenesis, and immune evasion (Miao et al., 2013). LDHA also plays an important role in T-cell differentiation. Aerobic glycolysis is a hallmark of activated T cells. LDHA promotes T-cell activation, proliferation, and migration (Xu et al., 2021a). LDHA deficiency leads to the defective expansion and differentiation of CD8^+^ T cells (Xu et al., 2021b). An in-depth explanation of the potential function of LDHA in the transition from NAFLD-cirrhosis to HCC within the immune microenvironment at the single-cell level remains unclear.

In the present study, we compared LDHA expression levels with clinical characteristics to validate their predictive accuracy and efficacy. We validated the performance of LDHA using multiple HCC datasets. Immunohistochemistry and flow cytometry were performed to validate the efficacy of LDHA. The investigation of LDHA in the immune microenvironment and its potential impact on the efficacy of immunotherapeutic interventions will offer new perspectives on the treatment of NAFLD-related LC progressing to HCC.

## 2 Materials and methods

### 2.1 Materials

LDHA (ET1608-57) antibody was purchased from HUABIO (Hangzhou HuaAn Biotechnology Co. Ltd., China). Rabbit anti-IgG (H + L) secondary antibody and FITC (#3003) were purchased from Report Biotech (Shijiazhuang, China). The CD3^+^ antibody was purchased from BD Biosciences (New York, NY, United States). General-type secondary antibody (PV-6000) and the DAB kit (ZLI-9018) were purchased from ORIGene (Beijing, China).

#### 2.1.1 Clinical blood and pathological tissue samples collection

Blood and pathological tissue samples were obtained from the Fifth Hospital of Shijiazhuang. In total, six and 17 peripheral blood samples were collected from healthy and HCC patients, respectively. Twenty-two HCC tissue samples were obtained from patients at the Fifth Hospital of Shijiazhuang between 2017 and 2022. These samples included the tumor and adjacent paracancerous tissue. None of the patients underwent systemic or local treatment prior to surgery. Detailed clinical data of the 22 patients, including sex, age, stage of liver cancer, Edmondson grade, cirrhosis grade, etiology, and viral replication, are shown in Supplementary Table S1. Twenty-two patients had virus-related HCC. Pathological tissue samples from two healthy individuals and four NAFLD-cirrhosis patients were collected from 2017 to 2020. This study was approved by the Medical Ethics Committee of the Fourth Hospital of Hebei Medical University and the Fifth Hospital of Shijiazhuang.

### 2.2 Methods

#### 2.2.1 Acquisition and processing of single-cell transcriptome data

We obtained raw scRNA-seq data from two cirrhotic liver samples with NAFLD and five healthy samples. Data were acquired from the Gene Expression Omnibus (GEO) under dataset GSE136103 (https://www.ncbi.nlm.nih.gov/gds). Quality control procedures were conducted within the R environment (version 4.1.2) following standard single-cell processing steps. To handle the count matrix, we utilized the Seurat package (version 4.0.4), specifically the Read10X function, to convert it into the “dgCMatrix” format. Individual objects are integrated into a collective object using the merge function, and the RenameCell function provides unique cell labels. To enhance the integrity of our data analysis, we implemented specific criteria to filter out cells of lower quality. We excluded genes expressed in fewer than three cells and removed cells expressing fewer than 200 genes. To standardize gene expression levels across cells, we applied global-scaling normalization using the “LogNormalize” method with a scaling factor of 10,000. For subsequent analysis, we focused on the top 2000 genes exhibiting the greatest variability in expression, identified using the FindVariableFeatures function. To mitigate any undesirable variations, such as unique molecular identifiers and mitochondrial content percentage, we utilized the ScaleData function with the “vars.to.regress” option. To streamline the complexity of our dataset, we performed principal component analysis (PCA) and selected the first 30 principal components (PCs) for further analysis. We applied the harmony method (Korsunsky et al., 2019) to counteract any batch effects between samples. We then visualized the cells in a lower-dimensional space using the uniform manifold approximation and projection method, which effectively preserved the local structure of the data. Clustering analysis was conducted based on the connectivity between cells using a shared nearest-neighbor graph generated through the Louvain algorithm. We systematically adjusted the resolution parameter within the range of 0.1–1 in the FindClusters function to identify the most suitable clustering resolutions. Evaluation of clustering trees at various resolutions using the clustree function led us to select a resolution of 0.5, resulting in clear and meaningful outcomes. We utilized the FindAllMarkers function to identify markers that were differentially expressed across the resulting clusters, employing a default nonparametric Wilcoxon rank-sum test with a Bonferroni correction. Cell annotation was performed using cell surface markers, established genes from relevant literature, and information from the CellMarker database (http://xteam.xbio.top/CellMarker/) (Zhang et al., 2019).

#### 2.2.2 Pseudo-time analysis

We utilized the “monocle” package (version 2.24.1) to conduct pseudo-time analysis as described in our methodology (Trapnell et al., 2014). To initiate the analysis, we employed the NewCellDataSet function to generate a new monocle object using the transcript count data. To define the trajectory progress, we included signature genes expressed in a minimum of 10% of the cells within the dataset, with a significance level of *p* < 0.01, as calculated using the differentialGeneTest function. Next, we employed the reduceDimension function to reduce the dataset to two dimensions for further analysis. The OrderCell function was then applied to arrange the cells based on their gene expression profiles. Following the execution of the orderCells function, T-cell states 1, 2, 3, 4, and 5 became discernible. To identify genes characteristic of each differentiation state, we utilized the Seurat package’s FindAllMarkers function. A gene was considered differentially expressed if the absolute value of the log2 fold change (|log2FC|) exceeded 0.5 and the adjusted *p*-value was less than 0.05.

#### 2.2.3 Acquisition and pre-processing of bulk transcriptome data

Transcriptome data were obtained from The Cancer Genome Atlas (TCGA) website (https://portal.gdc.cancer.gov/). Fragments per kilobase million (FPKM) were converted to transcripts per million (TPM). The clinical data of all patients were downloaded for further analysis.

#### 2.2.4 Prognostic model building based on machine-learning integration framework

In our research, we utilized ten distinct machine-learning algorithms: random survival forest (RSF), elastic network (Enet), Lasso, Ridge, stepwise Cox, CoxBoost, partial least squares regression for Cox (plsRcox), supervised principal components (SuperPC), generalized boosted regression (GBM), and survival support vector machine (survival-SVM). These algorithms played different roles within our study framework. One algorithm was tasked with variable screening, while another was involved in constructing a prognostic signature. We combined these algorithms in a total of 81 different combinations. To evaluate the performance of each signature, we calculated Harrell’s concordance index (C-index). The signature with the highest average C-index value was identified as the optimal one. Following the calculation of the T-cell differentiation-related risk score (TDRS) for each patient using the predict function, we determined the optimal cutoff value for the TDRS using the surv_cutpoint function from the “survminer” package. Based on this cutoff value, patients were categorized into high- and low-TDRS groups.

#### 2.2.5 Immunohistochemical (IHC) staining

Primary and secondary antibodies were used to immunostain sections of tissues embedded in paraffin to measure protein expression. The liver samples were embedded in paraffin blocks and sectioned at a thickness of 4 μm on slides. The sections were deparaffinized in xylene, dehydrated using a graded alcohol series, and rehydrated in deionized water. Tissue sections were washed in PBS and incubated in 10 mM EDTA buffer (pH 6.0) at 100°C for 15 min. The primary antibody was incubated overnight at 4°C. Non-specific antigens were blocked for 20 min using the catalase enzyme, followed by incubating the secondary antibody for 30 min, and then DAB chromogenic staining was performed. Two seasoned pathologists examined each portion and performed immunological scoring without being aware of clinical information. Pathologists performed histology scoring: tissue sections were scored according to the degree of staining (0–3 divided into negative staining, light yellow, light brown, and dark brown) and positive range (1–4 divided into 0–25%, 26–50%, 51–75%, and 76–100%). The scores were added at the end of the experiment, and the results were compared.

#### 2.2.6 Flow cytometry

A 100-µL aliquot of peripheral blood was added to 2 µL of PE-CD3^+^, 1 µL of LDHA, and 1 µL of secondary antibody-FITC. The mixture was then incubated for 15 min at 20°C away from light. Next, 1 mL of red blood cell lysis solution was added, and the mixture was further incubated for 15 min. After centrifugation at 1,000 rpm for 5 min at 4°C, the top fluid was discarded, and cells at the bottom of the tubes remained. After washing with PBS, the cells at the bottom of the tubes were harvested for flow cytometry.

#### 2.2.7 Statistical analysis

All statistical analyses were performed using SPSS (version 23.0.1; SPSS Inc., Chicago, IL, United States) or R (version 4.1.2). A significance level of *p* < 0.05 was deemed statistically significant. Flow cytometry analysis was conducted using a BD FACS Aria and interpreted using FlowJo software version 10.

## 3 Results

### 3.1 Single-cell sequencing analysis reveals different T-cell differentiation states in patients with liver cirrhosis

**FIGURE 1:**
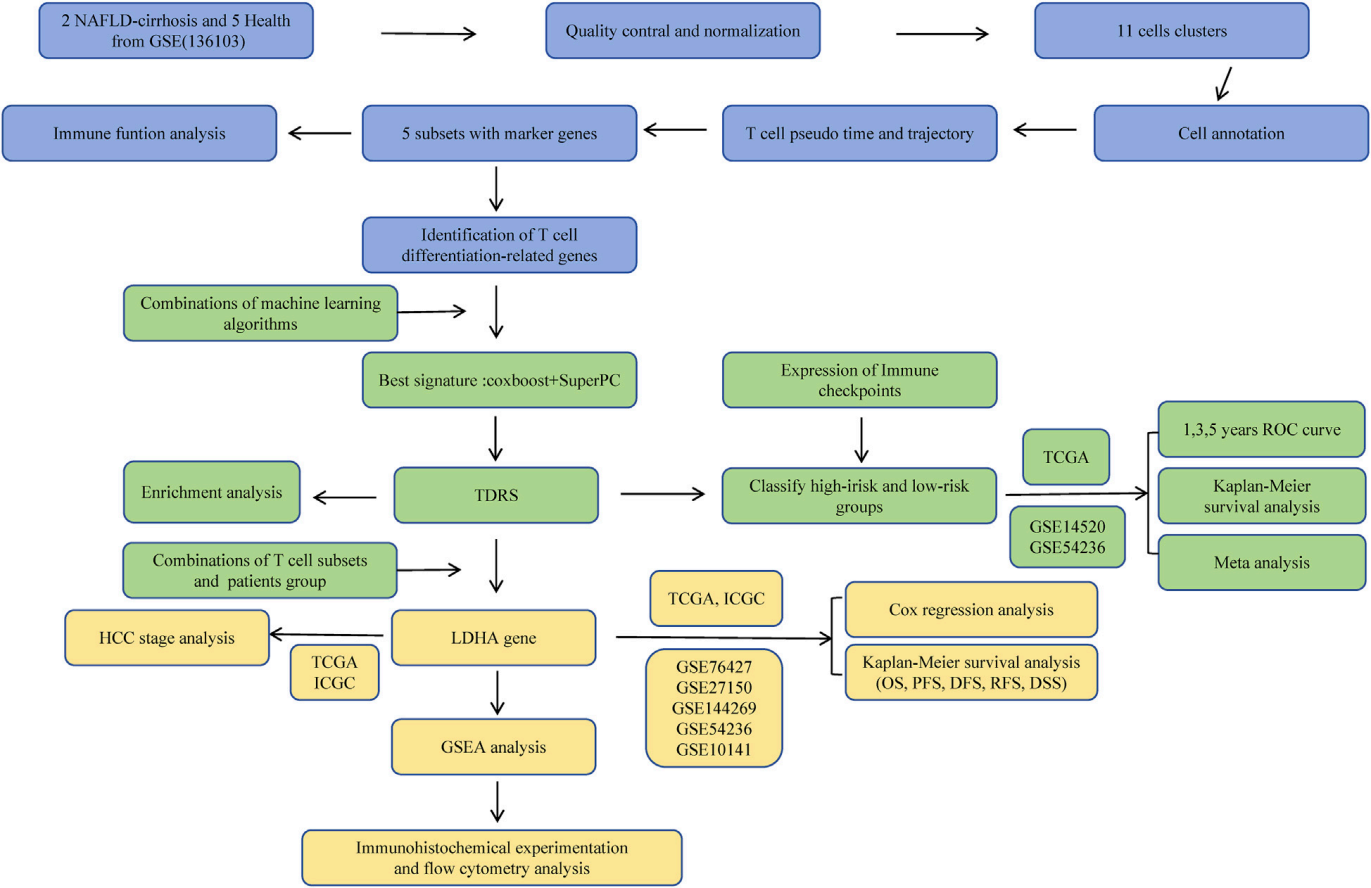
The workflow of this study.

**FIGURE 2:**
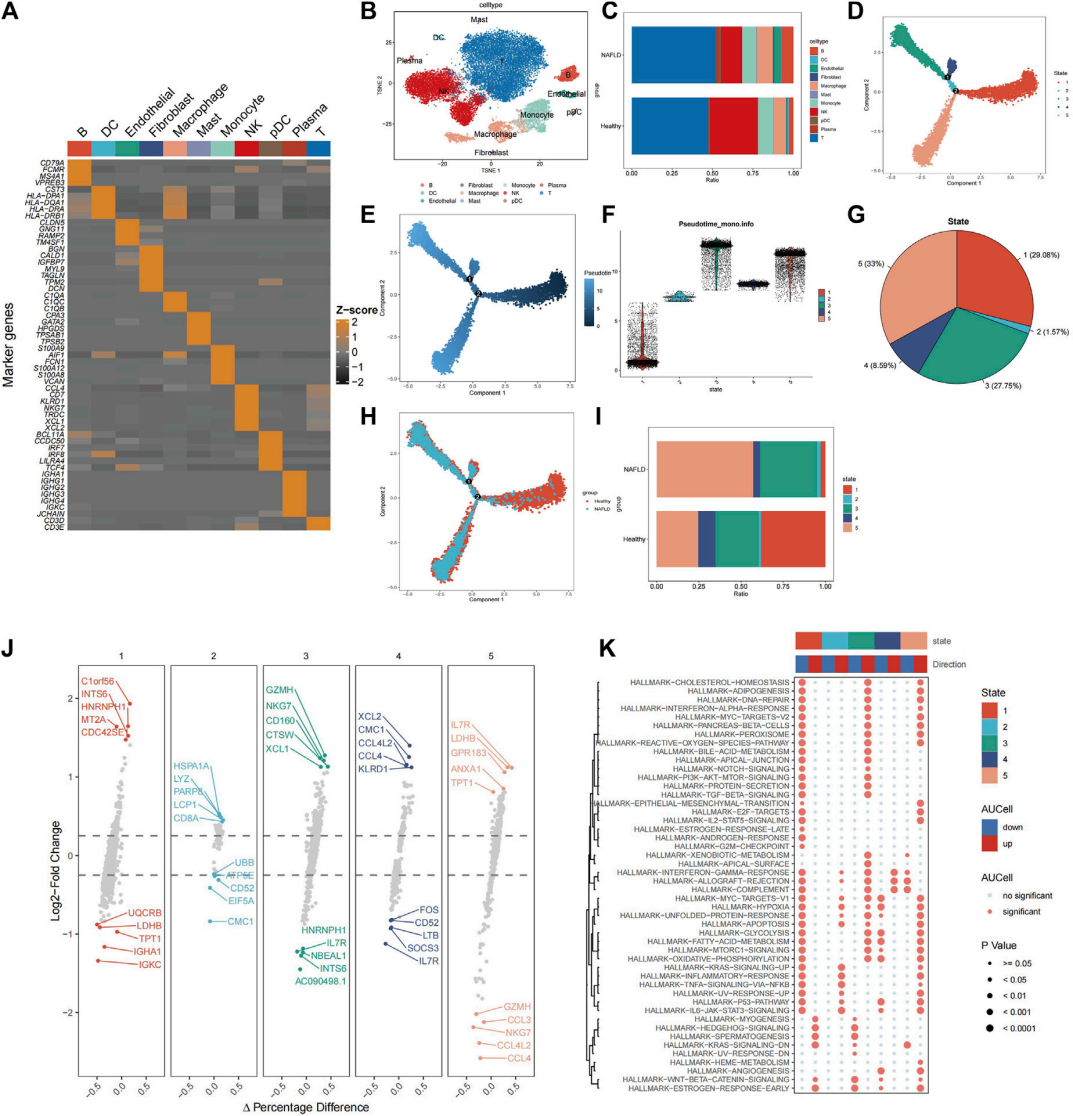
Identification of cells associated with NAFLD-cirrhosis phenotypes. **(A)** Expression of marker genes used in the cell annotation process. **(B)** The landscape of single-cell annotation. **(C)** Percentage of each cell type in the NAFLD-cirrhosis and healthy groups. **(D)** T cells were grouped into five subsets according to different differentiation states. **(E)** T cell differentiation trajectories. Darker colors indicate more bias toward the early stage of differentiation; lighter colors indicate more backward differentiation time sequences. **(F)** T cell differentiation trajectories. Lower positions indicate earlier differentiation degrees; higher positions mean later differentiation. **(G)** Percentage of each T cell differentiation state. **(H)** Identification of T cell differentiation states associated with cirrhosis phenotypes. **(I)** Percentage of T-cell differentiation states in different cohorts. **(J)** The top and the last five marked genes of each T-cell differentiation state. **(K)** Effector pathway activity in each of T-cell differentiation states.

The workflow of this study is illustrated in Figure 1. Healthy (n = 5) and liver cirrhosis samples (n = 2) were obtained from the GSE136103 dataset. After quality control, we obtained 7,199 single cells from two liver cirrhosis samples and 21,788 single cells from five healthy samples, which were clustered and labeled based on common cell surface marker genes and commonly referenced marker genes (Figure 2A). The results revealed that non-parenchymal cells were classified into 10 clusters: B cells, dendritic cells (DC), endothelial cells, fibroblast cells, macrophage cells, mast cells, monocytes, natural killer (NK) cells, p*lasmacytoid dendritic cells* (pDC), plasma cells, and T cells (Figure 2B). Because T cells constitute the largest cell subpopulation (Figure 2C), they were selected for further analysis in the subsequent study. Pseudo-time and trajectory analysis were employed to classify T cells into five subsets based on distinct differentiation states (Figure 2D). In Figure 2E, the colors represent the degree of differentiation, with darker colors indicating earlier stages of differentiation. The lower position of the T-cell subset in Figure 2F indicates an earlier degree of T-cell differentiation. We conclude that T cells undergo differentiation from state 1 to states 2, 4, and 5, as well as to state 3. Among these T cells, 29.08% were observed in state 1, 1.57% in state 2, 27.75% in state 3, 8.59% in state 4, and 33% in state 5 (Figure 2G). T cells from healthy individuals were mainly present in state 1, whereas T cells from NAFLD patients were mainly transformed to states 3 and 5 (Figure 2H). NAFLD patients exhibited a notable increase in T cells at stage 5 compared to healthy individuals, suggesting disease progression (Figure 2I). Subsequently, functional analysis was conducted to assess the impact of the five subsets of T cells. We identified the top five upregulated and downregulated genes within the different subsets (Figure 2J). Upregulation of IL7R, LDHB, GPR183, ANXA1, and TPT1 was observed in T cells in state 5. These upregulated genes play crucial roles in modulating diverse immunological responses, enhancing cell survival, and suppressing apoptosis. This is primarily achieved by activating the Janus kinase (JAK), transcription activator 5 (STAT5), and phosphatidylinositol 3-kinase (PI3K) signaling pathways (Zha et al., 2011; Daugvilaite et al., 2014; Mishra and Banerjee, 2019; Wang et al., 2022; Zhang et al., 2023). However, the top five downregulated genes in state 5 predominantly include members of the C-C motif chemokine ligand family (CCL3, CCL4L2, and CCL4), which augment tumor immunity by attracting lymphocytes and macrophages (Mukaida et al., 2020). A reduction in the expression of these chemokines impedes monocyte recruitment and facilitates immunosuppression.

Interestingly, the study found that the downregulated genes LDHB and TPT1 in T cells in state 1 were upregulated in state 5, suggesting that state 1 may have contrasting effects on pathways compared to state 5. Therefore, Gene Set Enrichment Analysis (GSEA) was used to perform functional analysis of different T-cell states based on differential gene expression. (Figure 2K). Red denotes activated pathways, whereas blue signifies inhibited pathways. Several pathways that were suppressed in state 1 were activated in states 3 and 5. In contrast to state 1, T cells in states 3 and 5 exhibited enhanced functionality in proinflammatory signaling pathways, such as the interferon-alpha response, TGF-BETA signaling, IL2-STAT5 signaling, inflammatory response, TNF signaling, and IL6-JAK-STAT3 signaling. In addition, some pathways such as interferon-gamma, allograft rejection, complement, myc-targets-v1, hypoxia, glycolysis, acid metabolism, oxidative phosphorylation, p53 pathway, and angiogenesis pathway were altered from states 1 to 3 and 5. The sequential progression of these pathways from repression to activation plays a vital role in disease development, suggesting that activation of these pathways promotes disease occurrence.

### 3.2 A prognostic signature linked to T-cell differentiation in NAFLD cirrhosis patients

**FIGURE 3:**
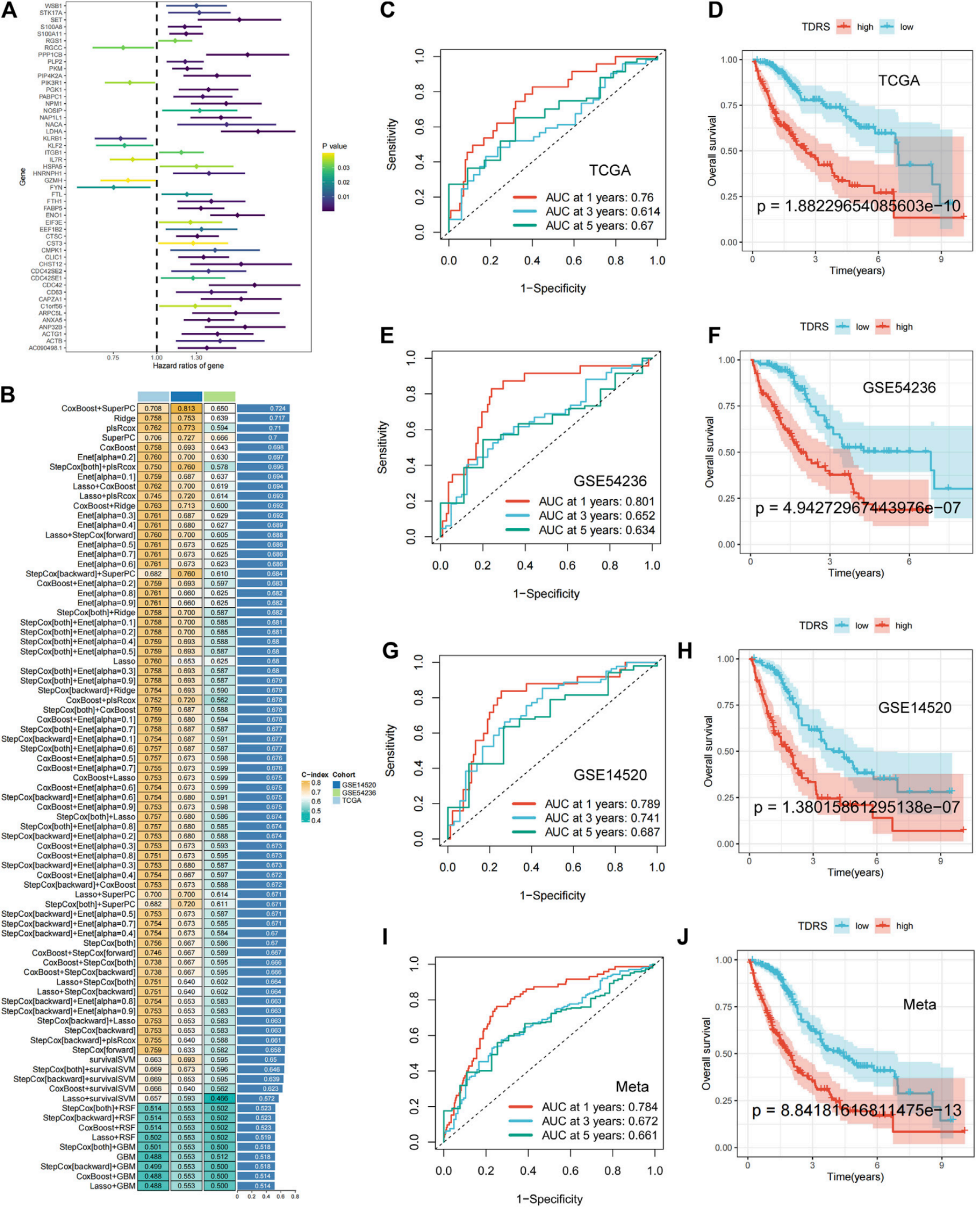
Machine-learning integration and the accuracy and validity of the prognostic model. **(A)** Univariate Cox regression identified 50 prognosis-related genes. **(B)** Eighty-one machine-learning integrated prognostic models and their C-index values were used to determine the CoxBoost + SuperPC signature with 18 genes as the best signature. **(C)** 1-year, 3-year, and 5-year ROC analysis in TCGA-LIHC. **(D)** OS analysis in TCGA-LIHC: poorer prognosis for high-risk patients than low-risk patients. **(E)** 1-year, 3-year, and 5-year ROC analysis in GSE54236. **(F)** OS analysis in GSE54236. **(G)** 1-year, 3-year, and 5-year ROC analysis in GSE14520. **(H)** OS analysis in GSE14520. **(I, J)** Meta-analysis of the ROC and OS on three combined datasets.

To evaluate the variances among T-cell subsets, we compared differentially expressed genes across groups. Differential gene expression analysis was conducted to identify genes that were uniquely expressed in specific T-cell subsets compared to other subsets. This analysis was based on the criteria of |log2FC| > 0.5 and adjusted *p*-value < 0.05. We identified 262 differentially expressed T-cell differentiation-related genes (Supplementary Table S2). Prognostic information and transcriptomic data of patients with HCC from The Cancer Genome Atlas (TCGA) were analyzed. Through univariate Cox regression analysis, we identified 50 prognosis-related genes, comprising seven protective genes (HR < 1) and 43 risk genes (HR > 1) (Figure 3A).

Machine-learning integration was performed for these 50 genes. To build a more accurate prognostic risk model and evaluate the performance of the machine-learning models, we employed ten machine-learning algorithms: random survival forest (RSF), elastic net (Enet), Lasso, Ridge, stepwise Cox, CoxBoost, partial least squares regression for Cox (plsRcox), supervised principal components (SuperPC), generalized boosted regression (GBM), and survival support vector machine (survival-SVM). These algorithms were applied to TCGA-LIHC datasets and two external validation sets (GSE 14520 and GSE 54236). When one algorithm was used to screen the variables, another was employed to construct a prognostic risk model. Harrell’s concordance index (C-index) was calculated for all validation datasets, and the signature with the highest average C-index was considered optimal. A total of 81 model combinations were successfully fitted for the analysis. The CoxBoost + SuperPC signature, comprising 18 genes (Supplementary Table S3), demonstrated the highest mean C-index value of 0.724, suggesting a superior prognostic predictive performance (Figure 3B).

Utilizing the CoxBoost + SuperPC combination, a risk score was computed for each case, denoted as the TDRS. We then analyzed the risk scores in the TCGA-LIHC, GSE54236, and GSE14520 datasets to evaluate the effectiveness of the prognostic models. Patients were categorized into high-risk and low-risk cohorts according to their individual risk scores utilizing the “srvminer” package for the identification of the most suitable threshold value. In TCGA-LIHC, the receiver operating characteristic (ROC) curve analysis of overall survival (OS) showed area under the curve (AUC) values of 0.76, 0.614, and 0.67 at 1 year, 3 years, and 5 years, respectively (Figure 3C). Two additional datasets (GSE54236 and GSE14520) were used to validate the prognostic impact. In addition, the AUC values for risk scores were 0.801, 0.652, 0.634 and 0.789, 0.741, and 0.687 at 1 year, 3 years, and 5 years in both the GSE54236 and GSE14520 datasets. (Figures 3E, G). Kaplan–Meier (*K–M*) analysis showed that high-risk patients in the TCGA-LIHC cohort had significantly poorer OS than low-risk patients (Figure 3D). Similarly, high-risk patients in the GSE54236 and GSE14520 datasets showed worse OS than low-risk patients, demonstrating the effectiveness and accuracy of our prognostic model (Figures 3F, H). When we combined the three datasets, we reached the same conclusion: AUC values of 0.71, 0.655, and 0.661 were obtained at 1 year, 3 years, and 5 years, respectively (Figure 3I), and high-risk patients exhibited a lower OS rate than low-risk patients, as illustrated in Figure 3J. The findings indicated that the TDRS consistently exhibited high performance levels across various groups.

### 3.3 Characterization of high- and low-risk groups and screening of hub gene by integrating the single‐cell and bulk-seq datasets

**FIGURE 4:**
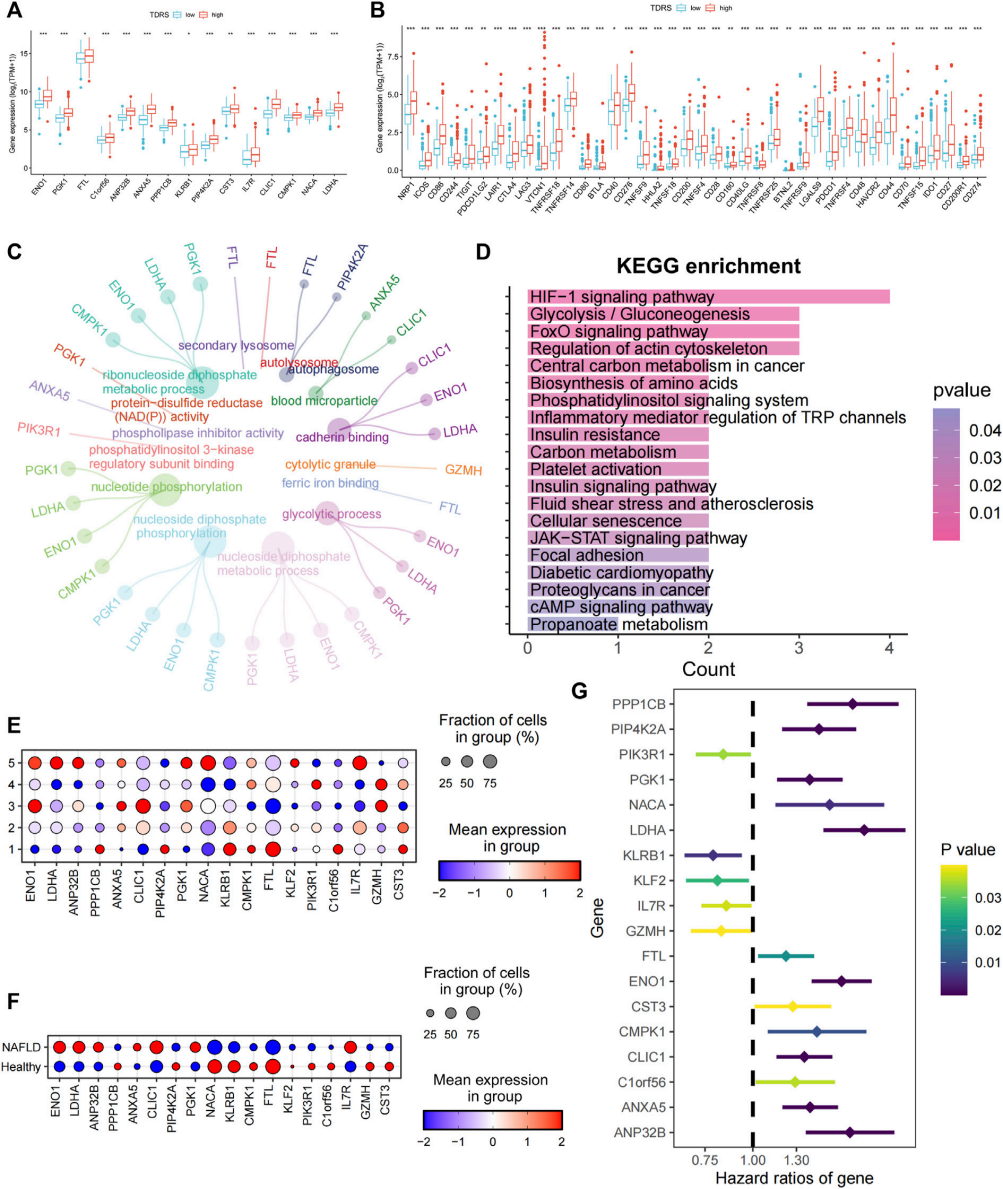
Characterization of high- and low-risk patients and examination of 18 genes. **(A)** Significantly differentially expressed profiles of the 14 signature genes in low- and high-risk groups. Low-risk patients are shown in blue, and high-risk patients are shown in red. **(B)** Analysis of correlations between low- and high-risk groups and immune checkpoints. **(C)** GO enrichment analysis. **(D)** KEGG enrichment analysis. **(E)** Expression of 18 genes in five different T cell states of single-cell data. **(F)** Expression of 18 genes in different cohorts. **(G)** Univariate Cox regression identifying 18 prognosis-related genes.

Subsequently, we examined the expression of 18 genes in both the high- and low-risk groups. Among the 18 T-cell differentiation-related genes analyzed, 14 genes, namely, ENO1, PGK1, FTL, C1orf56, ANP 32 B, ANXA5, PPP1CB, KLRB1, PIP4K2A, CST3, IL7R, CLIC1, CMPK1, NACA, and LDHA, exhibited significantly elevated expression levels in the high-risk group, as illustrated in Figure 4A. Next, the expression levels of 40 immune checkpoints, such as CTLA4, CD276, and CD274, were notably elevated in the high-risk group compared to those in the low-risk group. Divergence in the expression of immune-blocking sites between the high- and low-risk groups indicated a close association with immune activity. This suggests that individuals in the high-risk groups might be sensitive to immunotherapy (Figure 4B).

Enrichment analysis was conducted on differentially expressed genes between high- and low-risk patients. The biological process analysis identified gene ontology (GO) terms related to “nucleotide phosphorylation” and the “glycolytic process.” Furthermore, the GO items “autophagosome” and “blood microparticle” exhibited the highest level of enrichment in terms of cellular components. The predominant GO term related to molecular function (MF) was cadherin-binding activity, as illustrated in Figure 4C. Enrichment analysis based on the Kyoto Encyclopedia of Genes and Genomes (KEGG) (Figure 4D) revealed significant enrichment of pathways, including the HIF-1 signaling pathway, glycolysis/gluconeogenesis, the central carbon metabolism in cancer, biosynthesis of amino acids, the phosphatidylinositol signaling system, and inflammatory mediator regulation of TRP channels. Collectively, these findings indicate a strong correlation between these genes and phosphorylation, autophagy, and energy metabolism.

To investigate the roles of the genes in the five unique T-cell states using scRNA-seq, we analyzed the expression profiles of these genes in various T-cell subsets. Figure 4E shows a bubble plot illustrating the expression of these genes in each T-cell state. LDHA, ANP32B, NACA, and IL7R were predominantly expressed in T cells in state 5. We assessed the expression of these genes in both healthy individuals and patients with cirrhosis. ENO1, LDHA, ANP32B, CLIC1, and IL7R were highly expressed in patients with NAFLD cirrhosis (Figure 4F). The preceding analysis indicated a close association between T-cell status and the progression of cirrhosis. This suggests that LDHA and IL7R may play crucial roles in the progression of NAFLD cirrhosis. Furthermore, through univariate Cox regression analysis of the 18 genes, LDHA emerged as the most significant risk gene, whereas IL7R exhibited a protective effect (Figure 4G). Additionally, LDHA is highly expressed in T-cell state 5. The findings of this study indicate that LDHA plays a role in the progression of NAFLD-related cirrhosis to HCC and is linked to an unfavorable prognosis in patients with HCC.

### 3.4 LDHA showed a stable prognostic value

**FIGURE 5:**
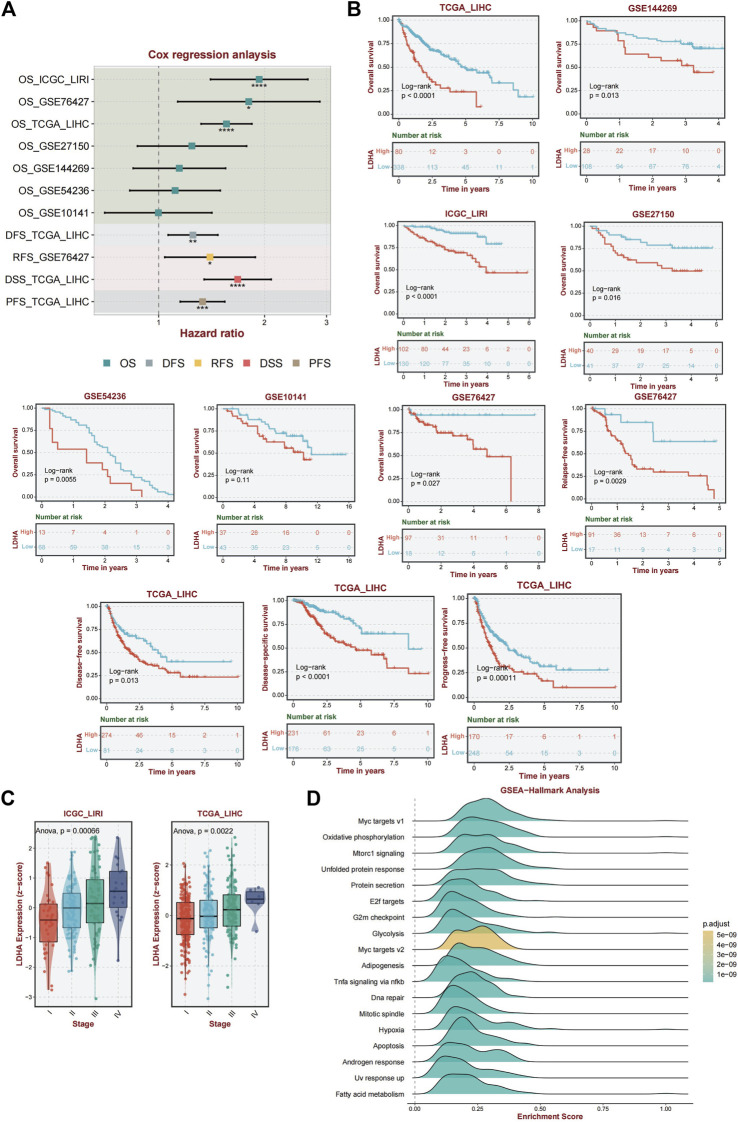
Analysis of the diagnostic and prognostic value of LDHA. **(A)** Univariate Cox regression analysis of LDHA in TCGA_LIHC, GSE144269, LCGC_LIRI, GSE27150, GSE54236, GSE1014, and GSE76427. **(B)** A K–M curve was used to display the 5-year OS rate of LDHA in different datasets. DFS of LDHA in TCGA_LIHC. DSS of LDHA in TCGA_LIHC. PFS of LDHA in TCGA_LIHC. RFS of LDHA in GSE76427. **(C)** LDHA expression level among different tumor stages in ICGC_LIRI and TCGA_LIHC data. **(D)** Activated pathway of LDHA using GSEA analysis.

Univariate Cox regression analysis was performed to validate LDHA as a causal gene in HCC. We analyzed the ICGC_LIRI, GSE76427, GSE27150, GSE144269, GSE54236, and GSE1014 datasets. Among these databases, ICGC_LIRI, GSE76427, GSE27150, GSE144269, and GSE1014 were all from Asian Yellow populations, and GSE54236 was from a European Caucasian population. LDHA was found to be a pathogenic factor influencing OS in patients with HCC (Figure 5A). In addition, LDHA acted as a hazard factor and significantly influenced progression-free survival (PFS), disease-free survival (DFS), and disease-specific survival (DSS) in patients with TCGA_LIHC. Similarly, LDHA was correlated with recurrence-free survival (RFS) in patients with hepatocellular carcinoma (HCC) in the GSE76427 database (Figure 5A).

Furthermore, the association between LDHA and survival was evaluated by performing a *K–M* survival curve analysis in these databases. OS was significantly longer for patients in the low LDHA expression group than for patients in the high LDHA expression group in TCGA_LIHC, GSE144269, ICGC_LIRI, GSE27150, GSE54236, GSE10141, and GSE76427 (Figure 5B). The same result was shown that the high LDHA expression had a worse PFS, DFS, and DSS in TCGA_LIHC patients and a worse RFS in the GSE76427 database (Figure 5B). In general, LDHA has shown consistent prognostic value for HCC, and LDHA overexpression may be associated with disease progression in patients with HCC.

Based on data from two distinct databases (ICGC_LIRI and TCGA_LIHC), a comparative analysis of LDHA mRNA expression levels in liver cancer tissues across various stages was conducted. Tukey’s honestly significant difference (HSD) method was used for *post hoc* testing. In the ICGC-LIRI data, there were significant differences between stage IV and stages I and II. In the TCGA data, there were significant differences between stages III and I. The results are presented in Supplementary Table S4. The findings revealed a significant correlation between LDHA expression and tumor stage, indicating that elevated LDHA expression levels are indicative of more advanced tumor stages (Figure 5C).

Given the important role of LDHA in immunity and the interesting results we obtained, we performed an enriched gene set pathway analysis of the role of LDHA dysregulation. Using GSEA (www.broad.mit.edu/gsea/), the top 18 different activated pathways (Figure 5D) in which the *p*-values were less than 0.01 were revealed corresponding to the LDHA mainly involved in Myc targets, oxidative phosphorylation, mTORC1 signaling, unfolded protein response, protein secretion, E2f targets, G2m checkpoint, glycolysis, adipogenesis, TNF-a signaling via NF-kb, DNA repair, mitotic spindle, hypoxia, apoptosis, androgen response, UV response up, and fatty acid metabolism signaling. GSEA analysis demonstrated that several inflammation-related, energy metabolism-related, and cancer-associated pathways were hyperactivated under high LDHA expression. These findings indicate a potential mechanism whereby the upregulation of LDHA contributes to metabolic disorders and proinflammatory responses, consequently affecting the prognostic outcomes of individuals with NAFLD that progresses to HCC.

### 3.5 Validation of LDHA in clinical samples

**FIGURE 6:**
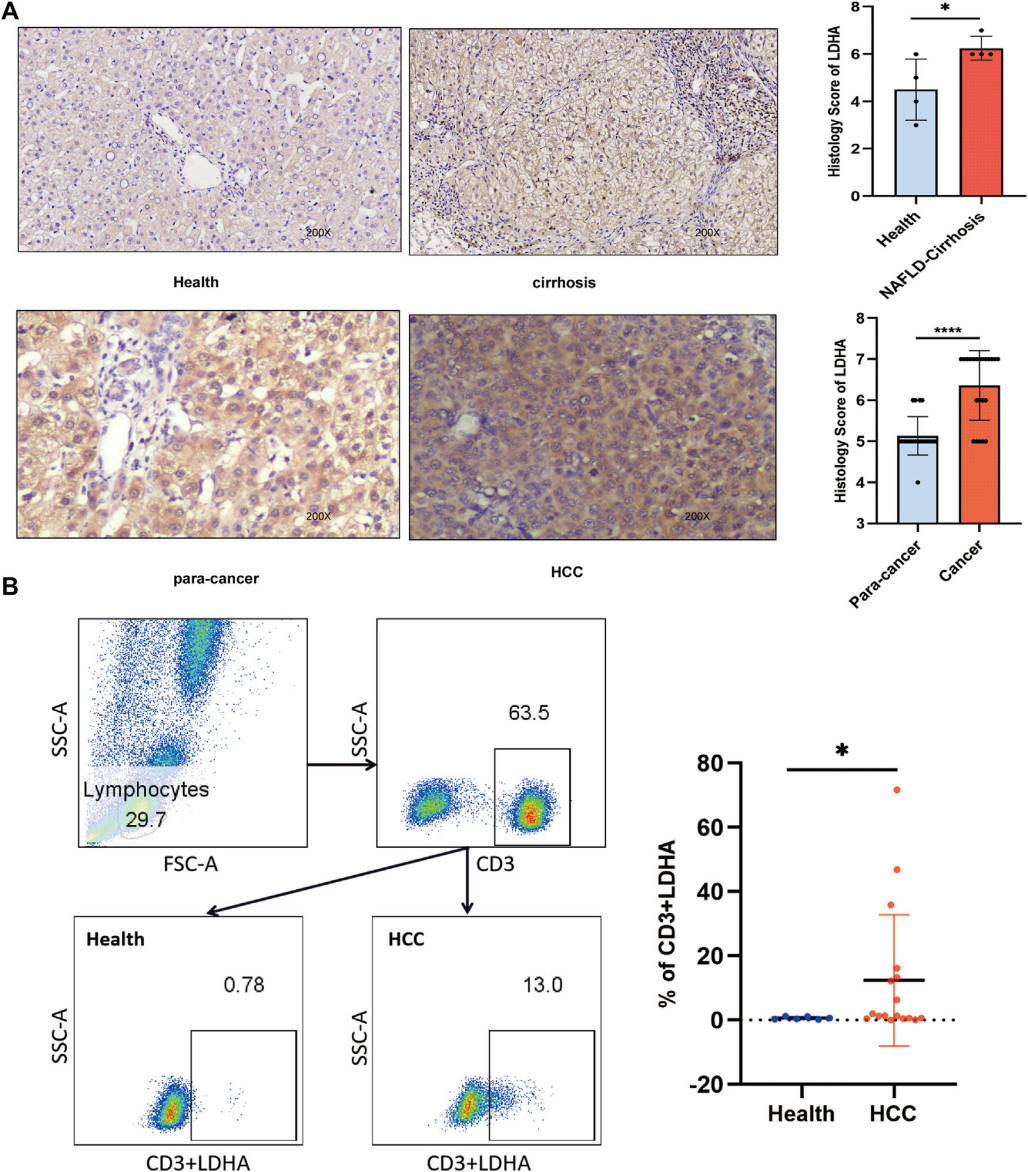
LDHA was used to verify by IHC and flow cytometry analysis. **(A)** Left panel, representative images of LDHA immunohistochemical staining of healthy vs. NAFLD cirrhosis and para-cancer vs. HCC cells. Right panel: immunohistochemistry scores. **(B)** Flow cytometry analysis was used to analyze the expression of LDHA in the healthy vs. the HCC group. Student’s t-test was used to analyze data. *p < 0.05; ****p < 0.0001.

To confirm the reliability of our results, we performed immunohistochemical (IHC) staining of healthy, NAFLD-cirrhotic, and HCC pathological tissue sections. Immunohistochemical scores based on the staining intensity and range were assessed by two qualified pathologists. LDHA expression was significantly higher in cancer tissues than in the corresponding adjacent tissues, as observed by IHC (*p* < 0.0001) (Figure 6A). Similarly, LDHA in NAFLD-cirrhosis samples was also markedly overexpressed compared to that in the healthy groups (*p* = 0.045) (Figure 6A). Flow cytometry was used to analyze LDHA expression in CD3^+^ cells in the peripheral blood of healthy individuals and patients with HCC. The gating strategies for the CD3+LDHA subsets are shown in Figure 6B. Compared with the peripheral blood of healthy individuals, the expression of CD3^+^ cells in LDHA was notably upregulated in the peripheral blood of patients with HCC (*p* = 0.044). These results were consistent with those of scRNA-seq and bulk-seq, indicating the accuracy and validity of our bioinformatics analysis and the significance of LDHA in NAFLD cirrhosis and HCC.

## 4 Discussion

HCC represents the most prevalent liver malignant neoplasm worldwide. On the one hand, due to changes in people’s diets and lifestyles, the incidence of NAFLD and NAFLD-related HCC has markedly increased (Younossi et al., 2016). Emerging tumor immunotherapy methods have recently greatly changed the treatment prospects of HCC, but the efficacy in HCC patients varies greatly; there are still many patients who fail to benefit from immunotherapy (Duffy et al., 2017; Xie et al., 2019). In contrast to most other malignancies, which are prototypical inflammation-related cancers, more than 80% of HCC cases are associated with cirrhosis (El-Serag, 2011; Shlomai et al., 2014; Walker et al., 2016). Immune evasion is one of the features that occurs during the initiation and evolution of HCC. The precancerous microenvironment (PME) plays an essential role in liver cirrhosis (Affo et al., 2017).

The liver is an immune-tolerant organ and is characterized by a highly immunosuppressive microenvironment. This restricts hepatic inflammatory responses, thereby mitigating autoimmune damage that may arise from persistent immune activation and exposure to antigens (Fu et al., 2019). Concurrently, this protective mechanism facilitates immune tolerance toward tumor-associated antigens and HCC (Makarova-Rusher et al., 2015). Enhanced immune suppression occurs because of continuous inflammatory cytokine production and recruitment of immune cells during the progression of liver disease. During LC, the immune functions of the liver are significantly compromised (Tsochatzis et al., 2014), and persistent inflammation and damage exacerbate cirrhosis and even lead to tumorigenesis (Albillos et al., 2022). A tolerant liver immunological network is amplified in HCC.

Single-cell sequencing techniques coupled with next-generation sequencing have been used to further define the pathogenesis of PME in cirrhosis. We categorized immune cells into healthy and NAFLD cirrhosis groups and observed a significant association between the cirrhosis group and T cells. T cells represent a predominant subset of lymphocytes within the immune system and are essential for orchestrating the adaptive immune response. CD4^+^ T cells have been implicated in the pathogenesis of NAFLD through the secretion of proinflammatory cytokines (Her et al., 2020). Th17 cells, a subset of CD4^+^ T cells, have been shown to exacerbate liver inflammation and fibrosis by secreting IL-17 cytokines (Meng et al., 2012). Regulatory T cells (Tregs) represent a subset of helper T cells distinguished by the expression of CD4^+^CD25 highFoxp3+. Tregs inhibit the activation of various immune cell types and trigger metabolic dysregulation associated with obesity (Sakaguchi et al., 2008). CD8^+^ T cells undergo dynamic changes during the progression of nonalcoholic fatty liver disease to hepatocellular carcinoma. An elevation in activated cytotoxic CD8^+^ T cells was observed in the early stages of NAFLD (Ghazarian et al., 2017; Grohmann et al., 2018). However, as cirrhosis and HCC advance, there is a notable decrease in the infiltration of CD8^+^ T cells along with a reduction in bioactive granule molecules, and CD8^+^ T cells change from effector T cells to depleted T cells (Fu et al., 2007).

Using differentiation trajectory and pseudo-time analysis, we were able to comprehend the role of these T cells in relation to PME. T cells were divided into five states, of which state 5 was the most relevant for disease. The differentially expressed genes in state 5 were enriched in proinflammatory signaling, energy metabolism signaling, hypoxia, and oxidative signaling. These activated signaling pathways form an inflammatory environment and promote disease progression. Next, by screening prognostic models with multiple machine-learning methods and combining the data from single cells, we obtained a crucial gene, LDHA, which is crucial for altered T-cell status in cirrhosis and HCC.

As a crucial enzyme in glycolysis, LDHA contributes to the production of lactic acid and NAD, which are associated with various immunological processes (Yan et al., 2023). In cancer cells, the Warburg effect promotes tumorigenesis and immune evasion even more (Hsu and Sabatini, 2008). Lactate produced by LDHA contributes to tumor progression, angiogenesis, and immunosuppression and is believed to be a vital regulator of tumor development, maintenance, and metastasis (Chen et al., 2023). Glycolysis plays an important role in T-cell development, proliferation, and function (Xu et al., 2021b). Glycolysis significantly influences the growth, proliferation, and functionality of T cells (Peng et al., 2016). T cells change their response to antigenic stimuli when an organism is diseased. Naive T cells rely on oxidative phosphorylation, whereas effector T cells rely on glycolysis (Rathmell et al., 2000). This is typically followed by the modification of LDHA and a change in energy metabolism. In hypoxic environments, lactate is preferentially converted from pyruvate by LDHA, which helps T cells quickly satisfy their energy needs for activation and proliferation (Lunt and Vander Heiden, 2011; Urbańska and Orzechowski, 2019). Additionally, LDHA enhances the expression of effector T-cell factors (Xu et al., 2021b), which mediate immune metabolic reprogramming and alter T-cell function not only through the glycolysis pathway but also by producing 2-hydroxyglutarate (2HG) (Peng et al., 2016; Ansa-Addo et al., 2017; Xu et al., 2021a; Notarangelo et al., 2022). In NAFLD, activated T cells have been shown to be closely related to the progression of liver cirrhosis (Zhou et al., 2022), suggesting that the activation of T cells by LDHA may be part of the reason for the progression of liver cirrhosis.

In our analysis, the transition of some pathways, including glycolysis, myc, hypoxia, glycolysis, acid metabolism, and oxidative phosphorylation from repression to activation, coincided with the process of NAFLD cirrhosis, indicating the importance of these pathways in the disease course. Enrichment analysis of LDHA revealed the abovementioned pathways. These results demonstrate that LDHA is involved in the pathogenesis of cirrhosis and liver cancer in both mechanistic and functional studies. We hope to further analyze the mechanism by which LDHA in T cells regulates the progression of cirrhosis and the occurrence of liver cancer.

Finally, to illustrate the prognostic utility of LDHA in PME and HCC, we conducted a thorough bioinformatics analysis based on single-cell data from many external bulk RNA sequencing datasets. The clinical samples provided good support for these results. Our research offers a useful strategy for predicting liver cirrhosis and HCC progression and treatment. However, the results of our study were not perfect. To further demonstrate the stability of the model, a larger cohort is required to confirm the established TDRS. Combining the TDRS with more comprehensive clinical characteristics could further enhance its prognostic prediction capabilities. Additional fundamental research is required to validate the regulatory role of LDHA in the development of cirrhosis in patients with HCC.

## Data Availability

The datasets presented in this study can be found in online repositories. The names of the repository/repositories and accession number(s) can be found in the article/Supplementary material.

## Abbreviations

CAID, Cirrhosis-associated immune dysfunction; C-index, Concordance index; DFS, Disease-free survival; DSS: Disease-specific survival; GBM, Generalized boosted regression; GEO, Gene expression omnibus; GO, Gene ontology; HCC, Hepatocellular carcinoma; KEGG, Kyoto Encyclopedia of Genes and Genomes; LC, Liver cirrhosis; LDHA, Lactate dehydrogenase A; NAFLD, Nonalcoholic fatty liver disease; OS: Overall survival; PCA, Principal component analysis; PFS, Progression-free survival; plsRcox, Partial least squares regression for Cox; PME, Precancerous microenvironment; RFS, Recurrence-free survival; ROC, Receiver operating characteristic; SuperPC, Supervised principal components; Survival-SVM, Survival support vector machine; TCGA, The Cancer Genome Atlas Program; TDRS, T-cell differentiation-related genes; Tregs, Regulatory T cells.
