# Supplementary material for: Single-cell sequencing and multiple machine learning algorithms to identify key T-cell differentiation gene for progression of NAFLD cirrhosis to hepatocellular carcinoma
Source: Front Mol Biosci. 2024 Jun 27;11:1301099. doi: 10.3389/fmolb.2024.1301099 (PMC11237165; doi:10.3389/fmolb.2024.1301099)
Supplement: Supplementary file 1 [file Table1.DOCX]

**Table S1：**Clinical features of 22 HCC patients.

| ID | Sex | Age | HCC stage | Child-Pugh  classification | Edmondson  Grades | Etiology | virus  replication |
| --- | --- | --- | --- | --- | --- | --- | --- |
| 1 | M | 60 | Ⅱb | B | moderately | HBV | No |
| 2 | M | 48 | Ⅰa | A | moderately | HBV  /HCV | No |
| 3 | F | 55 | Ⅰa | B | moderately-poorly | HBV | No |
| 4 | F | 64 | Ⅰa | B | moderately | HCV | Have |
| 5 | F | 54 | Ⅰb | B | moderately-poorly | HBV  /HCV | HCV virus  replication |
| 6 | M | 62 | Ⅰa | A | moderately | HBV | No |
| 7 | F | 52 | Ⅰa | A | moderately | HBV | No |
| 8 | M | 63 | Ⅰa | A | moderately | HBV | No |
| 9 | M | 53 | Ⅰa | A | highly-  moderately | HBV | No |
| 10 | M | 41 | Ⅱa | A | moderately | HBV | No |
| 11 | M | 64 | Ⅰa | B | moderately | HBV | No |
| 12 | M | 59 | Ⅰa | B | moderately | HCV | No |
| 13 | M | 63 | Ⅰa | A | moderately | HCV | No |
| 14 | M | 37 | Ⅰa | A | moderately | HBV | No |
| 15 | M | 56 | Ⅰa | B | moderately | HCV | Have |
| 16 | F | 62 | Ⅰa | A | moderately-poorly | HBV | No |
| 17 | M | 65 | Ⅰa | A | moderately | HBV | No |
| 18 | F | 75 | Ⅰa | B | moderately | HBV | No |
| 19 | M | 57 | Ⅰa | A | moderately-poorly | HBV | No |
| 20 | M | 51 | Ⅰa | A | highly | HBV | No |
| 21 | M | 55 | Ⅰa | A | moderately | HBV | No |
| 22 | M | 41 | Ⅰa | A | moderately | HBV | No |

**Note：**F:Female M:Male

The *HCC* stage was determined using Guidelines for Diagnosis and Treatment of Primary Liver Cancer (2022 Edition) in the China liver cancer staging system.(1)

The severity of cirrhosis in HCC patients was graded according to the Child-Pugh classification.

1. Zhou J, Sun H, Wang Z, Cong W, Zeng M, Zhou W, et al. Guidelines for the Diagnosis and Treatment of Primary Liver Cancer (2022 Edition). Liver cancer. 2023;12(5):405-44.<http://dx.doi.org/10.1159/000530495>
