# Supplementary material for: Single-cell sequencing and multiple machine learning algorithms to identify key T-cell differentiation gene for progression of NAFLD cirrhosis to hepatocellular carcinoma
Source: Front Mol Biosci. 2024 Jun 27;11:1301099. doi: 10.3389/fmolb.2024.1301099 (PMC11237165; doi:10.3389/fmolb.2024.1301099)
Supplement: Supplementary file 2 [file Table2.DOCX]

**Table S2:** 262 T-cell differentiation-related genes with different T cells cluster.

|  | p_val | avg_log2FC | pct.1 | pct.2 | p_val_adj | cluster | gene |
| --- | --- | --- | --- | --- | --- | --- | --- |
| 1 | 2.97E-304 | -0.757134291 | 0.7 | 0.965 | 6.42E-300 | 1 | ACTG1 |
| 2 | 1.70E-302 | 1.100924266 | 0.79 | 0.768 | 3.68E-298 | 1 | NBEAL1 |
| 3 | 1.08E-295 | -0.599700353 | 0.975 | 0.999 | 2.34E-291 | 1 | ACTB |
| 4 | 5.08E-293 | -1.162833493 | 0.071 | 0.42 | 1.10E-288 | 1 | IGHA1 |
| 5 | 3.00E-292 | -0.915169831 | 0.263 | 0.706 | 6.49E-288 | 1 | LDHB |
| 6 | 7.59E-285 | -0.821180301 | 0.42 | 0.832 | 1.64E-280 | 1 | VIM |
| 7 | 8.45E-260 | -0.655347262 | 0.592 | 0.944 | 1.83E-255 | 1 | MYL12A |
| 8 | 8.76E-244 | -0.720508153 | 0.241 | 0.663 | 1.90E-239 | 1 | ENO1 |
| 9 | 1.94E-227 | -0.638422512 | 0.511 | 0.905 | 4.19E-223 | 1 | CORO1A |
| 10 | 1.36E-219 | -0.634057415 | 0.361 | 0.798 | 2.95E-215 | 1 | MYL12B |
| 11 | 1.16E-210 | -0.615920249 | 0.225 | 0.616 | 2.51E-206 | 1 | CD53 |
| 12 | 5.48E-205 | -0.636061385 | 0.143 | 0.481 | 1.19E-200 | 1 | PGK1 |
| 13 | 5.09E-204 | -0.775301141 | 0.266 | 0.632 | 1.10E-199 | 1 | CTSW |
| 14 | 8.34E-204 | 0.552360465 | 0.956 | 0.986 | 1.80E-199 | 1 | FTL |
| 15 | 1.23E-194 | -0.603102917 | 0.341 | 0.727 | 2.65E-190 | 1 | ANXA1 |
| 16 | 8.69E-190 | -0.561034497 | 0.185 | 0.538 | 1.88E-185 | 1 | TRAF3IP3 |
| 17 | 1.78E-186 | -0.603544287 | 0.624 | 0.928 | 3.85E-182 | 1 | EEF1B2 |
| 18 | 3.70E-185 | -0.66537496 | 0.141 | 0.45 | 8.02E-181 | 1 | APOBEC3G |
| 19 | 5.63E-181 | -0.58570882 | 0.397 | 0.791 | 1.22E-176 | 1 | CD48 |
| 20 | 4.10E-180 | -0.59979671 | 0.102 | 0.386 | 8.88E-176 | 1 | ANXA6 |
| 21 | 6.48E-173 | -0.651561814 | 0.132 | 0.426 | 1.40E-168 | 1 | ITM2A |
| 22 | 1.73E-171 | -0.550645341 | 0.487 | 0.875 | 3.74E-167 | 1 | NPM1 |
| 23 | 1.91E-171 | -0.593810983 | 0.157 | 0.467 | 4.14E-167 | 1 | PLP2 |
| 24 | 1.71E-167 | -0.528550099 | 0.342 | 0.738 | 3.71E-163 | 1 | RAC2 |
| 25 | 8.65E-164 | -0.501607962 | 0.243 | 0.596 | 1.87E-159 | 1 | GIMAP7 |
| 26 | 3.78E-163 | 1.933691445 | 0.333 | 0.165 | 8.18E-159 | 1 | C1orf56 |
| 27 | 8.18E-160 | -0.502769347 | 0.264 | 0.622 | 1.77E-155 | 1 | COTL1 |
| 28 | 1.23E-158 | -0.506295648 | 0.153 | 0.452 | 2.66E-154 | 1 | ANP32B |
| 29 | 2.80E-157 | -0.51166914 | 0.309 | 0.687 | 6.07E-153 | 1 | IL2RG |
| 30 | 4.26E-153 | -0.569034503 | 0.088 | 0.331 | 9.21E-149 | 1 | C8orf59 |
| 31 | 1.40E-152 | 1.523512587 | 0.456 | 0.323 | 3.03E-148 | 1 | INTS6 |
| 32 | 3.81E-152 | -0.523519847 | 0.113 | 0.376 | 8.25E-148 | 1 | ATP5F1 |
| 33 | 1.43E-150 | -0.501337406 | 0.172 | 0.473 | 3.09E-146 | 1 | PKM |
| 34 | 9.16E-145 | 1.646594362 | 0.432 | 0.301 | 1.98E-140 | 1 | HNRNPH1 |
| 35 | 1.63E-136 | -0.533650014 | 0.11 | 0.352 | 3.54E-132 | 1 | HSPA5 |
| 36 | 3.40E-136 | -0.54037677 | 0.064 | 0.273 | 7.36E-132 | 1 | ANXA5 |
| 37 | 1.55E-122 | -0.598661597 | 0.067 | 0.258 | 3.34E-118 | 1 | IGLC3 |
| 38 | 2.59E-106 | -0.603584815 | 0.765 | 0.915 | 5.61E-102 | 1 | CXCR4 |
| 39 | 1.86E-100 | 0.531971037 | 0.871 | 0.911 | 4.02E-96 | 1 | ZFP36L2 |
| 40 | 3.84E-61 | 1.475810768 | 0.286 | 0.202 | 8.32E-57 | 1 | CDC42SE1 |
| 41 | 1.11E-50 | 1.165196529 | 0.277 | 0.202 | 2.40E-46 | 1 | PPP1CB |
| 42 | 1.66E-49 | 0.93468062 | 0.466 | 0.481 | 3.59E-45 | 1 | ZNF90 |
| 43 | 3.23E-49 | 0.748132508 | 0.644 | 0.626 | 7.00E-45 | 1 | KLRB1 |
| 44 | 1.92E-48 | 1.244997113 | 0.327 | 0.268 | 4.16E-44 | 1 | PRDM1 |
| 45 | 1.18E-41 | 1.080273397 | 0.4 | 0.392 | 2.56E-37 | 1 | RP11-347P5.1 |
| 46 | 6.45E-25 | 0.872236942 | 0.448 | 0.514 | 1.40E-20 | 1 | CDC42 |
| 47 | 1.28E-21 | 0.660252451 | 0.549 | 0.656 | 2.77E-17 | 1 | NEAT1 |
| 48 | 1.42E-17 | 0.934086407 | 0.331 | 0.344 | 3.08E-13 | 1 | CAPZA1 |
| 49 | 3.80E-17 | 0.863246654 | 0.399 | 0.451 | 8.22E-13 | 1 | STK17B |
| 50 | 3.02E-14 | 0.903546621 | 0.272 | 0.267 | 6.53E-10 | 1 | ETS1 |
| 51 | 6.30E-14 | 0.642689765 | 0.419 | 0.444 | 1.36E-09 | 1 | AC092580.4 |
| 52 | 8.00E-09 | 0.623871239 | 0.447 | 0.559 | 0.000173157 | 1 | MIF |
| 53 | 4.43E-08 | 0.926151274 | 0.273 | 0.291 | 0.000958772 | 1 | TNRC6B |
| 54 | 5.16E-08 | 0.836682028 | 0.267 | 0.281 | 0.001117253 | 1 | PIP4K2A |
| 55 | 6.78E-07 | 0.629181422 | 0.346 | 0.39 | 0.014677848 | 1 | IFITM1 |
| 56 | 5.03E-294 | -1.235849882 | 0.724 | 0.794 | 1.09E-289 | 3 | NBEAL1 |
| 57 | 1.28E-291 | 0.679683861 | 0.881 | 0.492 | 2.78E-287 | 3 | CST7 |
| 58 | 1.23E-286 | -1.449251055 | 0.61 | 0.736 | 2.65E-282 | 3 | AC090498.1 |
| 59 | 6.46E-277 | 0.937752756 | 0.496 | 0.175 | 1.40E-272 | 3 | KLRD1 |
| 60 | 2.46E-258 | 0.738112606 | 0.594 | 0.241 | 5.32E-254 | 3 | PRF1 |
| 61 | 5.30E-253 | 0.769813578 | 0.957 | 0.762 | 1.15E-248 | 3 | CCL4 |
| 62 | 5.05E-251 | 0.89179817 | 0.789 | 0.455 | 1.09E-246 | 3 | GZMK |
| 63 | 1.09E-248 | 1.128531246 | 0.481 | 0.186 | 2.36E-244 | 3 | XCL1 |
| 64 | 4.18E-233 | 0.781055581 | 0.261 | 0.057 | 9.04E-229 | 3 | FASLG |
| 65 | 8.89E-233 | 0.715872039 | 0.551 | 0.228 | 1.92E-228 | 3 | CTSC |
| 66 | 1.99E-232 | 0.72004039 | 0.432 | 0.145 | 4.31E-228 | 3 | LAG3 |
| 67 | 2.91E-231 | 0.847894285 | 0.423 | 0.147 | 6.29E-227 | 3 | CXCR6 |
| 68 | 4.15E-225 | 0.552628531 | 0.964 | 0.758 | 8.98E-221 | 3 | HCST |
| 69 | 1.03E-220 | 0.539307312 | 0.989 | 0.849 | 2.24E-216 | 3 | ACTG1 |
| 70 | 3.26E-215 | -0.566937693 | 0.985 | 0.975 | 7.05E-211 | 3 | FTL |
| 71 | 2.11E-211 | 0.796848966 | 0.512 | 0.213 | 4.56E-207 | 3 | CD8B |
| 72 | 2.44E-210 | 0.626667985 | 0.559 | 0.237 | 5.29E-206 | 3 | PYHIN1 |
| 73 | 1.74E-208 | 0.684505192 | 0.539 | 0.234 | 3.76E-204 | 3 | IGHA1 |
| 74 | 1.70E-196 | 0.612165105 | 0.385 | 0.134 | 3.67E-192 | 3 | MATK |
| 75 | 1.83E-190 | -1.2240705 | 0.417 | 0.604 | 3.97E-186 | 3 | IL7R |
| 76 | 2.65E-189 | -0.589396179 | 0.992 | 0.963 | 5.73E-185 | 3 | FTH1 |
| 77 | 6.37E-189 | 0.781590803 | 0.312 | 0.097 | 1.38E-184 | 3 | FABP5 |
| 78 | 7.49E-188 | -0.754651963 | 0.891 | 0.902 | 1.62E-183 | 3 | ZFP36L2 |
| 79 | 2.81E-184 | 0.58582259 | 0.8 | 0.465 | 6.08E-180 | 3 | LSP1 |
| 80 | 5.93E-184 | 0.798936279 | 0.477 | 0.209 | 1.28E-179 | 3 | HOPX |
| 81 | 7.40E-184 | 0.857449827 | 0.352 | 0.126 | 1.60E-179 | 3 | HLA-DRB5 |
| 82 | 2.63E-182 | 0.768074511 | 0.435 | 0.178 | 5.68E-178 | 3 | TRGC2 |
| 83 | 3.30E-182 | 0.560905608 | 0.57 | 0.262 | 7.15E-178 | 3 | ARPC5L |
| 84 | 5.49E-176 | 0.679621539 | 0.524 | 0.242 | 1.19E-171 | 3 | IFNG |
| 85 | 3.08E-173 | 0.520149387 | 0.975 | 0.795 | 6.67E-169 | 3 | TRAC |
| 86 | 3.84E-167 | 0.547328072 | 0.849 | 0.536 | 8.31E-163 | 3 | RAC2 |
| 87 | 6.07E-166 | 0.778698914 | 0.722 | 0.445 | 1.31E-161 | 3 | CCL4L2 |
| 88 | 4.72E-165 | 0.51240077 | 0.901 | 0.606 | 1.02E-160 | 3 | CLIC1 |
| 89 | 1.28E-160 | 0.666941204 | 0.541 | 0.264 | 2.76E-156 | 3 | ITM2A |
| 90 | 1.44E-160 | 0.51439143 | 0.635 | 0.326 | 3.11E-156 | 3 | GZMM |
| 91 | 1.00E-155 | 0.562419762 | 0.6 | 0.311 | 2.17E-151 | 3 | CD63 |
| 92 | 3.53E-153 | 0.547086598 | 0.271 | 0.086 | 7.63E-149 | 3 | ABI3 |
| 93 | 1.02E-150 | 0.500755719 | 0.887 | 0.588 | 2.21E-146 | 3 | MYL12B |
| 94 | 1.74E-147 | 0.528616108 | 0.656 | 0.356 | 3.76E-143 | 3 | STK17A |
| 95 | 1.85E-136 | 0.756635041 | 0.327 | 0.133 | 4.01E-132 | 3 | NCR3 |
| 96 | 6.49E-134 | 0.502692695 | 0.791 | 0.495 | 1.41E-129 | 3 | IL2RG |
| 97 | 1.05E-115 | 0.922090385 | 0.317 | 0.14 | 2.27E-111 | 3 | XCL2 |
| 98 | 8.58E-110 | 0.526698175 | 0.527 | 0.291 | 1.86E-105 | 3 | HLA-DRB1 |
| 99 | 1.40E-109 | -0.696742468 | 0.718 | 0.774 | 3.04E-105 | 3 | LTB |
| 100 | 1.07E-105 | 0.521089058 | 0.834 | 0.645 | 2.33E-101 | 3 | DUSP2 |
| 101 | 3.15E-105 | -0.688167721 | 0.789 | 0.748 | 6.81E-101 | 3 | EEF2 |
| 102 | 3.17E-104 | -0.807293481 | 0.651 | 0.7 | 6.86E-100 | 3 | TSC22D3 |
| 103 | 9.32E-95 | 0.917051047 | 0.641 | 0.42 | 2.02E-90 | 3 | CCL3 |
| 104 | 3.10E-84 | 0.567179961 | 0.44 | 0.249 | 6.72E-80 | 3 | CMC1 |
| 105 | 3.22E-84 | 0.569395846 | 0.376 | 0.199 | 6.97E-80 | 3 | CCL3L3 |
| 106 | 1.34E-80 | -0.577174585 | 0.81 | 0.762 | 2.90E-76 | 3 | PABPC1 |
| 107 | 5.52E-77 | -1.275530361 | 0.284 | 0.391 | 1.19E-72 | 3 | INTS6 |
| 108 | 4.81E-54 | -0.871938912 | 0.452 | 0.487 | 1.04E-49 | 3 | ZNF90 |
| 109 | 2.66E-45 | -1.183633858 | 0.29 | 0.358 | 5.75E-41 | 3 | HNRNPH1 |
| 110 | 7.61E-40 | -0.828648894 | 0.263 | 0.345 | 1.65E-35 | 3 | MTRNR2L8 |
| 111 | 5.63E-36 | -0.649963847 | 0.288 | 0.169 | 1.22E-31 | 3 | MT1X |
| 112 | 1.16E-33 | -0.783786857 | 0.255 | 0.321 | 2.52E-29 | 3 | CST3 |
| 113 | 4.74E-31 | -0.606686716 | 0.626 | 0.435 | 1.03E-26 | 3 | MT2A |
| 114 | 3.39E-29 | -0.76067319 | 0.252 | 0.314 | 7.33E-25 | 3 | SOCS3 |
| 115 | 1.12E-28 | -0.829352494 | 0.242 | 0.303 | 2.43E-24 | 3 | FOSB |
| 116 | 9.81E-23 | -0.707571787 | 0.434 | 0.447 | 2.12E-18 | 3 | DNAJB1 |
| 117 | 1.10E-22 | -0.535150479 | 0.559 | 0.543 | 2.37E-18 | 3 | SAT1 |
| 118 | 1.38E-18 | -0.793721248 | 0.256 | 0.295 | 2.98E-14 | 3 | TNFAIP3 |
| 119 | 1.76E-16 | -0.646624768 | 0.443 | 0.433 | 3.81E-12 | 3 | STK17B |
| 120 | 2.39E-14 | -0.569976942 | 0.496 | 0.464 | 5.18E-10 | 3 | SLC2A3 |
| 121 | 3.54E-13 | -0.640180321 | 0.236 | 0.264 | 7.66E-09 | 3 | NOSIP |
| 122 | 6.89E-11 | -0.585086391 | 0.26 | 0.277 | 1.49E-06 | 3 | CMPK1 |
| 123 | 2.24E-09 | -0.584993655 | 0.333 | 0.335 | 4.85E-05 | 3 | MCL1 |
| 124 | 3.04E-09 | -0.818555958 | 0.362 | 0.351 | 6.58E-05 | 3 | CRIP1 |
| 125 | 9.06E-09 | -0.763808579 | 0.28 | 0.287 | 0.000196026 | 3 | PRDM1 |
| 126 | 1.02E-08 | -0.652346615 | 0.411 | 0.388 | 0.000220227 | 3 | RP11-347P5.1 |
| 127 | 4.38E-08 | -0.680803529 | 0.283 | 0.287 | 0.000947902 | 3 | TNRC6B |
| 128 | 1.13E-07 | -0.650290306 | 0.265 | 0.27 | 0.002443029 | 3 | ETS1 |
| 129 | 3.01E-183 | 0.89892431 | 0.987 | 0.813 | 6.51E-179 | 4 | CCL5 |
| 130 | 5.28E-138 | 1.143859692 | 0.95 | 0.803 | 1.14E-133 | 4 | CCL4 |
| 131 | 6.79E-124 | -0.784617817 | 0.983 | 0.97 | 1.47E-119 | 4 | TPT1 |
| 132 | 2.09E-112 | -0.536164836 | 1 | 0.999 | 4.53E-108 | 4 | EEF1A1 |
| 133 | 5.60E-105 | 0.67064764 | 0.922 | 0.649 | 1.21E-100 | 4 | NKG7 |
| 134 | 5.09E-97 | 1.122074482 | 0.502 | 0.242 | 1.10E-92 | 4 | KLRD1 |
| 135 | 1.71E-96 | -0.82591224 | 0.704 | 0.867 | 3.69E-92 | 4 | CD52 |
| 136 | 3.87E-92 | 0.754930651 | 0.809 | 0.523 | 8.38E-88 | 4 | GZMK |
| 137 | 2.43E-85 | 1.400064253 | 0.392 | 0.17 | 5.25E-81 | 4 | XCL2 |
| 138 | 3.31E-85 | 0.735530292 | 0.815 | 0.58 | 7.16E-81 | 4 | CST7 |
| 139 | 1.13E-77 | 0.894490556 | 0.508 | 0.26 | 2.45E-73 | 4 | GZMH |
| 140 | 1.56E-74 | 1.253421111 | 0.495 | 0.284 | 3.37E-70 | 4 | CMC1 |
| 141 | 3.97E-73 | -1.123644506 | 0.307 | 0.575 | 8.59E-69 | 4 | IL7R |
| 142 | 1.54E-60 | -0.58340344 | 0.844 | 0.911 | 3.32E-56 | 4 | S100A4 |
| 143 | 3.16E-60 | -0.916445037 | 0.622 | 0.772 | 6.84E-56 | 4 | LTB |
| 144 | 4.76E-59 | 0.544506015 | 0.869 | 0.636 | 1.03E-54 | 4 | GZMA |
| 145 | 1.95E-54 | 0.66657676 | 0.495 | 0.278 | 4.21E-50 | 4 | CD8B |
| 146 | 2.50E-50 | -0.598906737 | 0.799 | 0.844 | 5.41E-46 | 4 | EEF1B2 |
| 147 | 2.76E-50 | 1.155773049 | 0.669 | 0.508 | 5.96E-46 | 4 | CCL4L2 |
| 148 | 4.29E-50 | 0.573654465 | 0.601 | 0.366 | 9.28E-46 | 4 | CD8A |
| 149 | 1.95E-49 | 0.738469011 | 0.533 | 0.34 | 4.21E-45 | 4 | HLA-DRB1 |
| 150 | 1.08E-47 | 0.612769058 | 0.707 | 0.509 | 2.35E-43 | 4 | CTSW |
| 151 | 4.10E-46 | 0.607221702 | 0.909 | 0.816 | 8.88E-42 | 4 | CD74 |
| 152 | 8.85E-43 | -0.694998222 | 0.575 | 0.725 | 1.92E-38 | 4 | VIM |
| 153 | 8.59E-41 | 0.879500591 | 0.477 | 0.306 | 1.86E-36 | 4 | IFNG |
| 154 | 4.36E-34 | 0.538859137 | 0.603 | 0.452 | 9.44E-30 | 4 | HLA-DPB1 |
| 155 | 2.77E-33 | -0.929087464 | 0.147 | 0.311 | 6.00E-29 | 4 | SOCS3 |
| 156 | 1.65E-31 | -0.500641499 | 0.666 | 0.761 | 3.57E-27 | 4 | GLTSCR2 |
| 157 | 4.39E-30 | 0.92726908 | 0.583 | 0.472 | 9.51E-26 | 4 | CCL3 |
| 158 | 6.58E-30 | 0.577706371 | 0.586 | 0.449 | 1.42E-25 | 4 | HLA-DRA |
| 159 | 7.55E-30 | -0.593155885 | 0.558 | 0.667 | 1.63E-25 | 4 | IGKC |
| 160 | 2.72E-29 | -0.665507667 | 0.443 | 0.589 | 5.88E-25 | 4 | LDHB |
| 161 | 2.57E-28 | 0.592546099 | 0.377 | 0.237 | 5.56E-24 | 4 | TRGC2 |
| 162 | 2.04E-26 | 0.533325675 | 0.272 | 0.157 | 4.42E-22 | 4 | TIGIT |
| 163 | 2.05E-26 | -0.68080932 | 0.176 | 0.332 | 4.44E-22 | 4 | IGHA1 |
| 164 | 4.62E-26 | -0.820129711 | 0.461 | 0.597 | 9.99E-22 | 4 | FOS |
| 165 | 5.60E-26 | -0.562835167 | 0.441 | 0.593 | 1.21E-21 | 4 | S100A11 |
| 166 | 2.42E-25 | -0.687219041 | 0.509 | 0.643 | 5.23E-21 | 4 | KLRB1 |
| 167 | 5.25E-25 | 0.548336111 | 0.444 | 0.324 | 1.14E-20 | 4 | FYN |
| 168 | 1.51E-24 | 0.883878376 | 0.359 | 0.238 | 3.26E-20 | 4 | CCL3L3 |
| 169 | 1.59E-24 | 0.597686917 | 0.293 | 0.18 | 3.44E-20 | 4 | HLA-DQB1 |
| 170 | 1.62E-24 | 0.511686622 | 0.508 | 0.388 | 3.51E-20 | 4 | HLA-DPA1 |
| 171 | 1.02E-23 | 0.619377786 | 0.255 | 0.149 | 2.21E-19 | 4 | S100A8 |
| 172 | 2.08E-23 | -0.525782047 | 0.49 | 0.625 | 4.49E-19 | 4 | NAP1L1 |
| 173 | 4.30E-20 | -0.525946039 | 0.246 | 0.389 | 9.30E-16 | 4 | PLP2 |
| 174 | 4.52E-20 | -0.572673663 | 0.214 | 0.354 | 9.78E-16 | 4 | RGCC |
| 175 | 3.33E-19 | 0.517543991 | 0.323 | 0.219 | 7.21E-15 | 4 | PIK3R1 |
| 176 | 1.26E-18 | 0.500601362 | 0.463 | 0.366 | 2.72E-14 | 4 | CLEC2B |
| 177 | 2.78E-18 | -0.633825405 | 0.147 | 0.266 | 6.01E-14 | 4 | NOSIP |
| 178 | 4.07E-15 | 0.505841473 | 0.336 | 0.247 | 8.81E-11 | 4 | TYROBP |
| 179 | 8.36E-15 | 0.740987168 | 0.353 | 0.26 | 1.81E-10 | 4 | XCL1 |
| 180 | 7.46E-290 | 0.803923175 | 0.835 | 0.552 | 1.62E-285 | 5 | FXYD5 |
| 181 | 1.39E-289 | -2.388291104 | 0.361 | 0.602 | 3.01E-285 | 5 | CCL4L2 |
| 182 | 1.12E-279 | 0.794183712 | 0.923 | 0.678 | 2.42E-275 | 5 | LTB |
| 183 | 1.49E-275 | 0.706100523 | 0.911 | 0.689 | 3.22E-271 | 5 | NPM1 |
| 184 | 3.48E-274 | 1.05988884 | 0.403 | 0.13 | 7.53E-270 | 5 | GPR183 |
| 185 | 3.42E-254 | -1.784798962 | 0.087 | 0.351 | 7.40E-250 | 5 | KLRD1 |
| 186 | 5.91E-240 | 0.587763088 | 0.965 | 0.797 | 1.28E-235 | 5 | CD52 |
| 187 | 8.70E-230 | 0.75630204 | 0.874 | 0.633 | 1.88E-225 | 5 | VIM |
| 188 | 1.31E-220 | 0.59751354 | 0.921 | 0.667 | 2.84E-216 | 5 | UQCRB |
| 189 | 6.73E-214 | 0.633055043 | 0.895 | 0.683 | 1.46E-209 | 5 | GLTSCR2 |
| 190 | 6.72E-213 | 0.851127398 | 0.784 | 0.532 | 1.46E-208 | 5 | ANXA1 |
| 191 | 3.69E-197 | 0.530295641 | 0.969 | 0.833 | 7.98E-193 | 5 | NACA |
| 192 | 9.04E-195 | -0.676334179 | 0.796 | 0.825 | 1.96E-190 | 5 | HCST |
| 193 | 2.95E-193 | -0.98269552 | 0.387 | 0.627 | 6.39E-189 | 5 | GZMK |
| 194 | 2.26E-185 | 0.720184662 | 0.819 | 0.579 | 4.89E-181 | 5 | IGKC |
| 195 | 8.10E-175 | -1.463994389 | 0.172 | 0.394 | 1.75E-170 | 5 | IFNG |
| 196 | 3.59E-172 | 0.740359938 | 0.383 | 0.16 | 7.77E-168 | 5 | CD40LG |
| 197 | 1.31E-171 | 0.781863287 | 0.456 | 0.218 | 2.84E-167 | 5 | SOCS3 |
| 198 | 3.40E-170 | -1.172591826 | 0.145 | 0.371 | 7.35E-166 | 5 | CD8B |
| 199 | 3.76E-170 | 0.722946358 | 0.744 | 0.507 | 8.14E-166 | 5 | FOS |
| 200 | 1.74E-167 | 0.580417153 | 0.803 | 0.579 | 3.76E-163 | 5 | EIF3E |
| 201 | 9.61E-167 | 0.625980233 | 0.743 | 0.5 | 2.08E-162 | 5 | S100A11 |
| 202 | 1.43E-166 | 0.576681122 | 0.847 | 0.607 | 3.09E-162 | 5 | TSC22D3 |
| 203 | 4.18E-165 | 0.537408065 | 0.881 | 0.699 | 9.04E-161 | 5 | EEF2 |
| 204 | 1.49E-162 | 0.686469358 | 0.856 | 0.674 | 3.23E-158 | 5 | DUSP1 |
| 205 | 6.90E-160 | 0.739779371 | 0.401 | 0.185 | 1.49E-155 | 5 | NOSIP |
| 206 | 5.53E-157 | -1.130198662 | 0.197 | 0.409 | 1.20E-152 | 5 | PRF1 |
| 207 | 1.30E-148 | -2.121545255 | 0.382 | 0.53 | 2.81E-144 | 5 | CCL3 |
| 208 | 8.20E-148 | 0.75496656 | 0.493 | 0.267 | 1.77E-143 | 5 | RGCC |
| 209 | 7.78E-146 | 0.61007074 | 0.647 | 0.414 | 1.68E-141 | 5 | LDHA |
| 210 | 1.77E-145 | 0.546301964 | 0.766 | 0.538 | 3.83E-141 | 5 | NAP1L1 |
| 211 | 1.78E-143 | 0.669320132 | 0.306 | 0.123 | 3.85E-139 | 5 | TIMP1 |
| 212 | 2.49E-143 | -0.781934185 | 0.629 | 0.731 | 5.38E-139 | 5 | DUSP2 |
| 213 | 9.35E-141 | -0.932443067 | 0.432 | 0.572 | 2.02E-136 | 5 | CTSW |
| 214 | 2.19E-139 | -1.822565762 | 0.127 | 0.308 | 4.75E-135 | 5 | CCL3L3 |
| 215 | 9.25E-138 | -1.577930951 | 0.143 | 0.329 | 2.00E-133 | 5 | XCL1 |
| 216 | 7.35E-136 | 0.624210106 | 0.524 | 0.304 | 1.59E-131 | 5 | PLP2 |
| 217 | 1.42E-128 | 0.647839041 | 0.313 | 0.139 | 3.07E-124 | 5 | TRADD |
| 218 | 1.38E-119 | 0.653659422 | 0.592 | 0.379 | 2.98E-115 | 5 | BIRC3 |
| 219 | 1.73E-117 | 0.606944537 | 0.282 | 0.121 | 3.74E-113 | 5 | AQP3 |
| 220 | 2.80E-114 | 0.548483909 | 0.325 | 0.153 | 6.05E-110 | 5 | CCDC109B |
| 221 | 3.70E-114 | 0.646006399 | 0.285 | 0.124 | 8.02E-110 | 5 | ITGB1 |
| 222 | 1.05E-113 | 0.718069621 | 0.253 | 0.102 | 2.27E-109 | 5 | KLF2 |
| 223 | 2.30E-113 | -0.95732131 | 0.111 | 0.281 | 4.98E-109 | 5 | LAG3 |
| 224 | 7.19E-112 | -0.635867216 | 0.607 | 0.726 | 1.56E-107 | 5 | ID2 |
| 225 | 3.41E-111 | 0.518816177 | 0.424 | 0.234 | 7.39E-107 | 5 | RNASET2 |
| 226 | 1.71E-110 | 0.547921306 | 0.421 | 0.232 | 3.70E-106 | 5 | ARHGAP15 |
| 227 | 4.90E-109 | -0.688639507 | 0.821 | 0.825 | 1.06E-104 | 5 | CD74 |
| 228 | 1.50E-106 | -1.615003825 | 0.201 | 0.352 | 3.24E-102 | 5 | CMC1 |
| 229 | 2.44E-106 | -1.134383586 | 0.14 | 0.303 | 5.27E-102 | 5 | TRGC2 |
| 230 | 6.77E-103 | 0.503506046 | 0.493 | 0.302 | 1.46E-98 | 5 | ANP32B |
| 231 | 9.20E-101 | 0.579146647 | 0.92 | 0.847 | 1.99E-96 | 5 | CXCR4 |
| 232 | 5.67E-100 | 0.512433745 | 0.588 | 0.389 | 1.23E-95 | 5 | SNHG8 |
| 233 | 2.01E-88 | -0.876348605 | 0.225 | 0.377 | 4.35E-84 | 5 | PYHIN1 |
| 234 | 1.01E-81 | -0.934575604 | 0.265 | 0.401 | 2.18E-77 | 5 | HLA-DRB1 |
| 235 | 5.17E-73 | 0.514105327 | 0.29 | 0.159 | 1.12E-68 | 5 | IGLC3 |
| 236 | 1.64E-70 | -0.882594588 | 0.18 | 0.308 | 3.54E-66 | 5 | KLRG1 |
| 237 | 1.60E-69 | 0.560924159 | 0.649 | 0.485 | 3.46E-65 | 5 | JUN |
| 238 | 2.90E-63 | -0.600534998 | 0.347 | 0.481 | 6.29E-59 | 5 | AC092580.4 |
| 239 | 2.37E-57 | -1.350659091 | 0.426 | 0.519 | 5.13E-53 | 5 | MT2A |
| 240 | 6.43E-56 | -0.780790306 | 0.256 | 0.367 | 1.39E-51 | 5 | SH2D1A |
| 241 | 9.96E-54 | -0.669006724 | 0.344 | 0.445 | 2.16E-49 | 5 | GZMM |
| 242 | 8.86E-49 | -0.645185341 | 0.29 | 0.395 | 1.92E-44 | 5 | APOBEC3G |
| 243 | 5.22E-46 | -0.625995619 | 0.2 | 0.306 | 1.13E-41 | 5 | CHST12 |
| 244 | 1.21E-42 | -0.61104558 | 0.157 | 0.256 | 2.62E-38 | 5 | CXCR6 |
| 245 | 9.64E-41 | -0.693306158 | 0.289 | 0.376 | 2.09E-36 | 5 | RP5-1171I10.5 |
| 246 | 1.31E-37 | -0.607666569 | 0.258 | 0.347 | 2.84E-33 | 5 | CTSC |
| 247 | 5.18E-35 | -0.587763877 | 0.426 | 0.485 | 1.12E-30 | 5 | HLA-DPB1 |
| 248 | 2.24E-32 | -0.63968157 | 0.215 | 0.297 | 4.86E-28 | 5 | LINC00152 |
| 249 | 2.32E-32 | -0.59965158 | 0.281 | 0.36 | 5.02E-28 | 5 | FYN |
| 250 | 5.52E-30 | -0.54868576 | 0.298 | 0.372 | 1.19E-25 | 5 | ARPC5L |
| 251 | 1.65E-29 | -0.63217311 | 0.213 | 0.29 | 3.57E-25 | 5 | RUNX3 |
| 252 | 1.33E-28 | -0.906083216 | 0.298 | 0.36 | 2.89E-24 | 5 | HNRNPH1 |
| 253 | 3.30E-27 | -0.555592045 | 0.357 | 0.419 | 7.15E-23 | 5 | HLA-DPA1 |
| 254 | 6.53E-25 | -0.616297334 | 0.392 | 0.447 | 1.41E-20 | 5 | RGS1 |
| 255 | 8.24E-23 | -0.76492443 | 0.217 | 0.273 | 1.78E-18 | 5 | TYROBP |
| 256 | 2.18E-20 | -0.74353967 | 0.333 | 0.376 | 4.71E-16 | 5 | INTS6 |
| 257 | 4.77E-19 | -0.523618826 | 0.19 | 0.25 | 1.03E-14 | 5 | SYTL3 |
| 258 | 1.81E-18 | -0.578294839 | 0.24 | 0.295 | 3.92E-14 | 5 | PIP4K2A |
| 259 | 8.91E-18 | -0.717247899 | 0.253 | 0.301 | 1.93E-13 | 5 | PRDM1 |
| 260 | 7.98E-15 | -0.576767214 | 0.314 | 0.353 | 1.73E-10 | 5 | CAPZA1 |
| 261 | 4.34E-14 | -0.592094494 | 0.377 | 0.403 | 9.39E-10 | 5 | RP11-347P5.1 |
| 262 | 7.90E-12 | -0.561760135 | 0.647 | 0.624 | 1.71E-07 | 5 | KLRB1 |
