# Supplementary material for: Single-cell sequencing and multiple machine learning algorithms to identify key T-cell differentiation gene for progression of NAFLD cirrhosis to hepatocellular carcinoma
Source: Front Mol Biosci. 2024 Jun 27;11:1301099. doi: 10.3389/fmolb.2024.1301099 (PMC11237165; doi:10.3389/fmolb.2024.1301099)
Supplement: Supplementary file 3 [file Table3.DOCX]

**Table S3；**18 genes of the CoxBoost+SuperPC signature.

|  | features | algorithm |
| --- | --- | --- |
| 1 | ENO1 | CoxBoost+SuperPC |
| 2 | LDHA | CoxBoost+SuperPC |
| 3 | ANP32B | CoxBoost+SuperPC |
| 4 | PPP1CB | CoxBoost+SuperPC |
| 5 | ANXA5 | CoxBoost+SuperPC |
| 6 | CLIC1 | CoxBoost+SuperPC |
| 7 | PIP4K2A | CoxBoost+SuperPC |
| 8 | PGK1 | CoxBoost+SuperPC |
| 9 | NACA | CoxBoost+SuperPC |
| 10 | KLRB1 | CoxBoost+SuperPC |
| 11 | CMPK1 | CoxBoost+SuperPC |
| 12 | FTL | CoxBoost+SuperPC |
| 13 | KLF2 | CoxBoost+SuperPC |
| 14 | PIK3R1 | CoxBoost+SuperPC |
| 15 | C1orf56 | CoxBoost+SuperPC |
| 16 | IL7R | CoxBoost+SuperPC |
| 17 | GZMH | CoxBoost+SuperPC |
| 18 | CST3 | CoxBoost+SuperPC |
