# Supplementary material for: Single-cell sequencing and multiple machine learning algorithms to identify key T-cell differentiation gene for progression of NAFLD cirrhosis to hepatocellular carcinoma
Source: Front Mol Biosci. 2024 Jun 27;11:1301099. doi: 10.3389/fmolb.2024.1301099 (PMC11237165; doi:10.3389/fmolb.2024.1301099)
Supplement: Supplementary file 4 [file Table4.DOCX]

**Table S4:**Post hoc test and the difference between the groups in ICGC_LIRI and TCGA_LIHC.

ICGC_LIRI

|  | diff | lwr | upr | p adj |
| --- | --- | --- | --- | --- |
| II-I | 0.2647441 | -0.22050022 | 0.7499884 | 0.493361706 |
| III-I | 0.4523630 | -0.05894494 | 0.9636710 | 0.103476388 |
| IV-I | 0.9658766 | 0.27195864 | 1.6597946 | 0.002168002 |
| III-II | 0.1876190 | -0.18933208 | 0.5645700 | 0.571646426 |
| IV-II | 0.7011325 | 0.09931994 | 1.3029451 | 0.015044627 |
| IV-III | 0.5135136 | -0.10950506 | 1.1365322 | 0.145830692 |

TCGA_LIHC

|  | diff | lwr | upr | p adj |
| --- | --- | --- | --- | --- |
| II-I | 0.1692349 | -0.10468880 | 0.4431585 | 0.382449289 |
| III-I | 0.4466943 | 0.15438291 | 0.7390056 | 0.000565303 |
| IV-I | 0.4823193 | -0.52291257 | 1.4875511 | 0.602252393 |
| III-II | 0.2774594 | -0.05858759 | 0.6135064 | 0.145061047 |
| IV-II | 0.3130844 | -0.70572505 | 1.3318939 | 0.857229475 |
| IV-III | 0.0356250 | -0.98828148 | 1.0595315 | 0.999739401 |
